# [Corrigendum] Dl‑3‑n‑butylphthalide alleviates cognitive impairment in amyloid precursor protein/presenilin 1 transgenic mice by regulating the striatal‑enriched protein tyrosine phosphatase/ERK/cAMP‑response element‑binding pro‑​tein signaling pathway

**DOI:** 10.3892/etm.2022.11742

**Published:** 2022-11-30

**Authors:** Yan Zhao, Wen-Qiang Yang, Lu Yu, Jing Yang, Hai-Rong Zhu, Lin Zhang

Exp Ther Med 23:319, 2022; DOI: 10.3892/etm.2022.11248

Subsequently to the publication of the above article, an interested reader drew to the authors’ attention that, in Fig. 6C on p. 8, the data panels showing the ‘Hippocampus / APP/PS1’ and ‘Hippocampus / NBP 30 mg/kg’ experiments appeared to be overlapping, such that these may have been derived from the same original source.

The authors had retained acess to their original data, and were able to identify that the affected panels were inadvertently assembled incorrectly in this figure; specifically, the data panel for the ‘Hippocampus / APP/PS1’ experiment was chosen incorrectly. The revised version of Fig. 6 is shown on the next page, now featuring the correct data panel for the ‘Hippocampus / APP/PS1’ experiment. Note that this error did not have a major impact on either the overall results or on the conclusions reported in this study. The authors thank the interested reader for bringing this matter to their attention. All the authors agree with the publication of this corrigendum, and are grateful to the Editor of *Experimental and Therapeutic Medicine* for granting them the opportunity to publish this; furthermore, they apologize to the readership for any inconvenience caused.

## Figures and Tables

**Figure 6:**
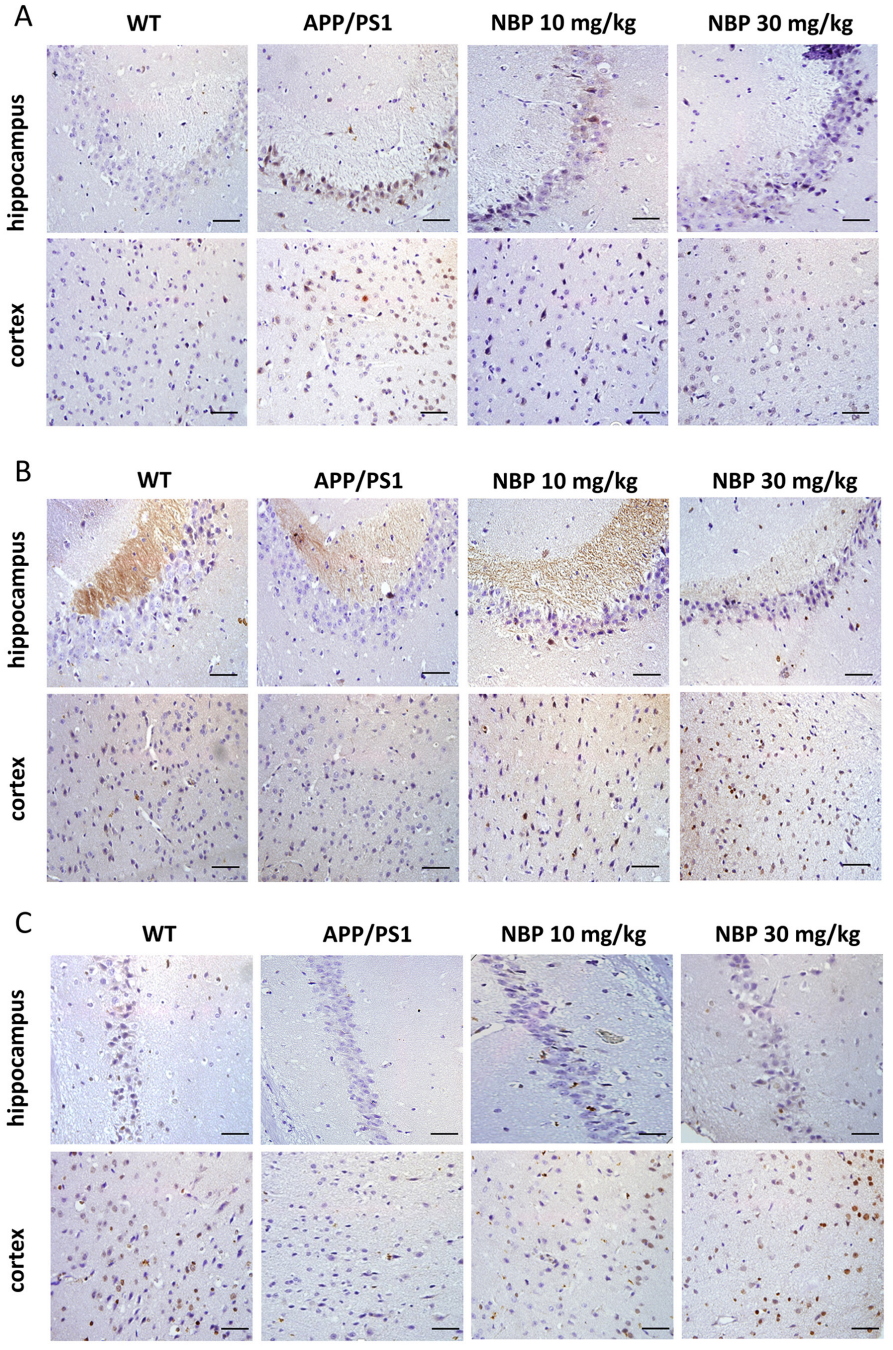
Effects of NBP on the expression of STEP61, p-ERK1/2 and p-CREB in APP/PS1 transgenic mouse brains. (A) Representative images of immunohistochemical staining of the STEP61 in the cortex and hippocampus. (B) Representative images of immunohistochemical staining of the p-ERK1/2 in the cortex and hippocampus. (C) Representative images of immunohistochemical staining of the p-CREB in the cortex and hippocampus. Brown indicates positive staining. Scale bars, 50 µm. NBP, Dl-3-n-butylphthalide; STEP, striatal-enriched protein tyrosine phosphatase; p-, phosphorylated; CREB, cAMP-response element-binding protein; WT, C57BL/6 group.

